# Transmembrane Transporter Sema3D Serves as a Tumor Suppressor in Localized Clear Cell Renal Cell Carcinoma

**DOI:** 10.1155/2022/3204189

**Published:** 2022-06-30

**Authors:** Ruiyang Xie, Jie Wu, Bingqing Shang, Chuanzhen Cao, Xingang Bi, Hongzhe Shi, Jianzhong Shou, Youyan Guan

**Affiliations:** Department of Urology, National Cancer Center/National Clinical Research Center for Cancer/Cancer Hospital, Chinese Academy of Medical Sciences and Peking Union Medical College, Chaoyang District, Beijing, China

## Abstract

**Background:**

The transmembrane transporter Sema3D is a vital molecule involved in axon guidance and carcinogenesis of variant malignancies. However, the relationship between Sema3D and clear cell renal cell carcinoma (ccRCC) is barely reported and remains unclear.

**Methods:**

Sema3D expression and the connection of clinical and histological characteristics were first analyzed with transcriptome data in the TCGA repository. We then located and examined the Sema3D expression in ccRCC patients by using immunofluorescence staining in the tissue microarray. The prognostic value of Sema3D in localized ccRCC was evaluated by Cox proportional hazard analysis. Functional and gene set enrichment analysis (GSEA), Gene Ontology (GO), and Kyoto Encyclopedia of Genes and Genomes (KEGG) were performed to describe the potential mechanisms of Sema3D in ccRCC. Correlation analysis between Sema3D and tumor-infiltrating lymphocytes was calculated by ssGSEA.

**Results:**

In 86 ccRCC patients, Sema3D mRNA and protein expression were downregulated in tumor tissues than the para-tumor tissues, and Sema3D was dominantly expressed in the extracellular space. Low expression of Sema3D was associated with advanced tumor stage, advanced histological grade, and poor prognosis in ccRCC. In the subgroup analysis of 81 localized ccRCC patients, Sema3D expression level was an independent protective prognostic factor for overall survival (OS) (HR = 0.125, *p*=0.043). Coagulation, complement, estrogen response, and KRAS signaling hallmark gene sets were identified as Sema3D-related signaling pathways. The expression level of Sema3D was significantly correlated with a high abundance of several immune cells (neutrophils, eosinophils, and *T* helper cells).

**Conclusions:**

Transmembrane transporter Sema3D is an efficient prognostic biomarker for localized ccRCC patients, by playing the role of tumor suppressor in ccRCC. Sema3D can be a novel therapeutic target for ccRCC.

## 1. Introduction

Renal cell carcinoma (RCC) is a common genitourinary malignancy worldwide. In 2021, as reported, the estimated new cases and deaths of cancer in kidneys were 76,080 and 13,780, respectively, in the USA [1]. Clear cell renal cell carcinoma (ccRCC) is the most frequent subtype of RCC that accounts for 80–90% of all patients [2]. Genetic mutation and expression play a fundamental role in oncogenesis of ccRCC. Some prominent mutated genes were detected and identified as driver mutations in the TRACERx renal cohort study, including VHL, PBRM1, SETD2, PTEN, and TP53 [3]. Overall, these driver mutations contain oncogenes and tumor suppressor genes, promoting the oncogenesis together by complex progress. The great mutational heterogeneity within ccRCC was revealed, but the integrative landscape of pathogenic gene mutation and expression has not been fully explored. Another recent research in our team completed whole-exome sequencing (WES) in 21 ccRCC samples [4] and then annotated single-nucleotide variants of semaphorin 3D (Sema3D) as a potential pathogenic driver mutation by PeCanPie [5].

Sema3D encodes a member of the semaphorin III family of secreted signaling proteins that are correlated with axon guidance during neuronal development. In terms of cancer, the underlying mechanisms of semaphorin signaling are more complicated. It was reported that Sema3D had strong anti-angiogenic effects in a glioma tumor model of mice, which suggested potential tumor suppressive function by inhibition of tumor angiogenesis of Sema3D [6]. Another study in pancreatic ductal adenocarcinoma also demonstrated that the axon guidance pathway mediated by Sema3D might be involved in inhibiting pancreatic tumor progression *in vivo* [7]. To date, however, no previous study has investigated the link between Sema3D and ccRCC. In this study, by validating the correlation between tumor Sema3D expression level and the prognosis of ccRCC patients, we identified the cancer suppressive role of Sema3D.

## 2. Materials and Methods

### 2.1. Patient Characteristics and Tumor Samples

**Figure 1:**
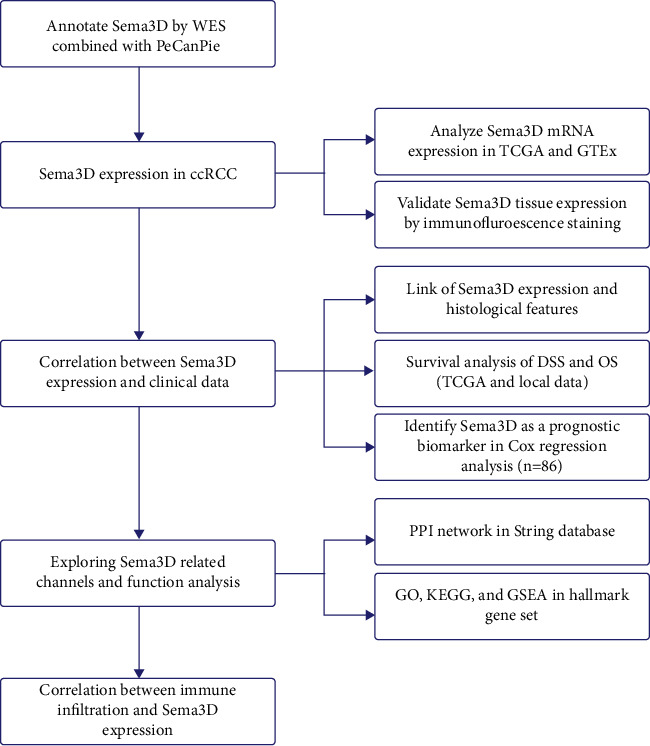
Overall workflow of the study.

For validation of Sema3D protein expression in the tumor, we obtained the tissue microarray (TMA) of 90 ccRCC patients (HKid-CRC180Sur-01, Shanghai Outdo Biotech, China). A total of 86 ccRCC tumor tissues and 86 paired para-tumor normal tissues were further analyzed. The grouping criteria for sample selection were as follows: 1. the tumor sample was histologically confirmed ccRCC. 2. An available survival time with outcome was noted (alive, dead, or deleted). 3. Complete clinical data (TNM grade and ISUP histologic grade). 4. One tumor sample and one paired para-tumor tissue sample were collected in the matching patient for the grouping of “tumor” and “para-tumor.” The exclusion rules were as follows: 1. the slice was stained with low quality. 2. The tissue missing area ratio was higher than 80%. Four patients were excluded for the low quality and large area of missing specimens. The clinical characteristics and histological information are presented in Table 1. This study was approved by local ethics committees (Shanghai Outdo Biotech, YB M-05-01). The overall workflow of this research is presented in Figure 1.

### 2.2. Immunofluorescence Staining

The TMA of ccRCC and para-tumor normal tissues were heated for 1 h in a dry oven at 60°C, and then deparaffinized and rinsed in water for 30 min at room temperature. Next, the TMA was incubated in 2.5% blocking solution with donkey serum (abs935, Absin Bioscience Inc, China). Immunofluorescence staining was performed to detect the expression of Sema3D proteins, according to the manufacturer's protocols. The slide was incubated with the primary antibody for Sema3D in a humidified chamber at 4°C overnight (recombinant rabbit polyclonal antibody, 1 : 50 dilution; HPA037522, Atlas Antibodies, Sweden). The sections were then incubated with secondary antibodies (Alexa Fluor 488, 1 : 200 dilution; 711-545-152, Jackson ImmunoResearch) for 2 h at room temperature. DAPI was used for nuclear staining (Boster Biological Technology, CA, US). Finally, the slides were analyzed with a confocal laser scanning microscope (Axio Scope A1, Zeiss, Germany). The Sema3D expression was determined as the area ratio of the stained field and the overall field. For Sema3D expression quantification, the area was measured and calculated in ImageJ software version 1.8.0 in each view of 70× magnification [8]. The diameter of each slice was approximately 1600 *μ*·m, and the entire slice area of one sample was analyzed for the expression quantification without random selection. The accuracy of measurements was confirmed by two independent investigators who were unaware of the patients' clinical information.

### 2.3. Bioinformatics Analysis

The RNAseq data of ccRCC in 631 samples (539 in tumor and 92 in normal tissue) with matched clinical profiles were downloaded from The Cancer Genome Atlas (TCGA) and Genotype-Tissue Expression Project (GTEx) repository (https://portal.gdc.cancer.gov/). To present the expression profile of Sema3D in pan-cancer, the GEPIA2 database was utilized (http://gepia2.cancer-pku.cn) [9]. Bioinformatics analysis was performed in *R* software 3.6.3, and the “ggplot2” package was used to investigate and visualize differential expression [10]. The differentially expressed genes (DEGs) were then analyzed with the “DESeq2” package, with the cut-off value set >75% in the high expression group and <25% in the low expression group (*p* < 0.05, |log2 fold change| ≥2) [11]. The Search Tool for the Retrieval of Interacting Genes Database (STRING database; https://string-db.org/) was used to create a protein-protein interaction (PPI) network [12]. Nodes with confidence of interactive score higher than 0.7 were included for network. We calculated immune infiltration by ssGSEA in the “GSVA” package [13], and the matching cell markers were previously described [14]. Gene Ontology (GO) and Kyoto Encyclopedia of Genes and Genomes (KEGG) functional analyses were carried out using the Database for Annotation, Visualization and Integrated Discovery (DAVID; https://david.ncifcrf.gov) [15]. Gene set enrichment analysis (GSEA) was performed by using the “clusterprofiler” package [16]. For each GSEA, gene sets with |NES| >1, NOM *p* < 0.05, and FDR *q* < 0.25 were considered significant. The hallmark gene set was selected (h.all.v7.2.symbols.gmt) as the reference gene set.

### 2.4. Statistical Analysis

SPSS Statistics software version 23.0 and Prism software version 8.0.2 were used for the statistical analysis. X-tile software version 3.6.1 was used to confirm the cut-off value of the Sema3D expression level [17]. Patient characteristics were summarized by count and percentage for categorical variables, and Mann–Whitney *U* test was performed to compare the distribution of categorical data between different Sema3D expression sets. The Mann–Whitney *U* test was also used to analyze the expression level of Sema3D in tumor and normal tissue samples. The Kaplan–Meier method and log-rank test were applied to determine the survival analysis. Disease-specific survival (DSS) was defined as the time elapsed between date of diagnosis with RCC and date of death from RCC. Overall survival (OS) was defined as the time from nephrectomy to death or last follow-up. We computed the log-rank *p* value and hazard ratio (HR) with 95% confidence intervals (CIs). Univariate and multivariate Cox regression models were utilized to evaluate the HRs of prognostic factors. For immune infiltration, we used the Spearman correlation coefficient to assess the correlation of gene expression. All statistical analyses were evaluated at a two-sided *p* value of 0.05.

## 3. Results

### 3.1. Sema3D Is Low Expressed in ccRCC

**Figure 2:**
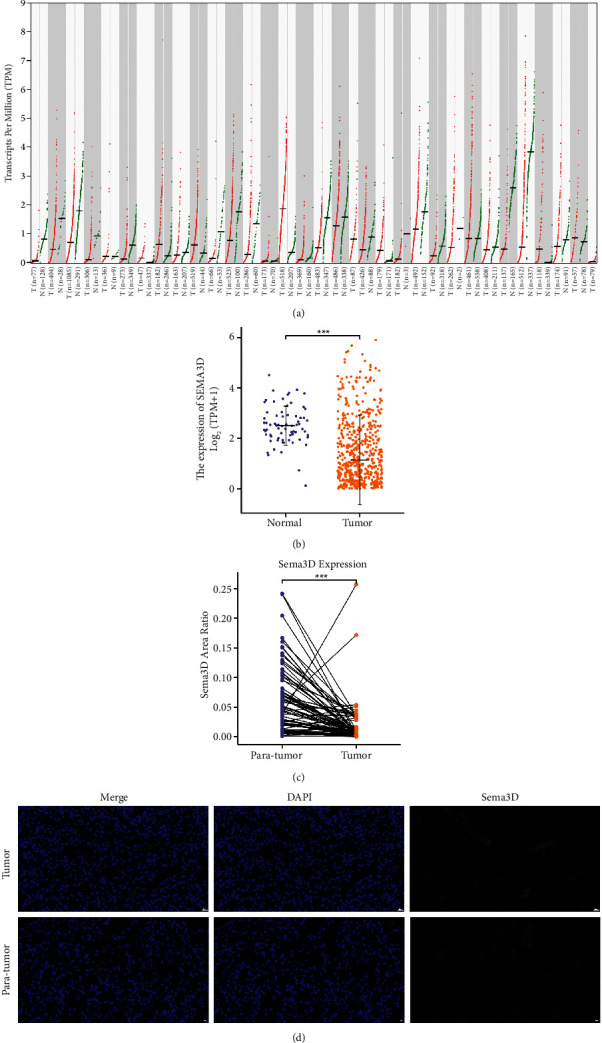
Expression of Sema3D in clear cell renal cell carcinoma (ccRCC). (a) Sema3D expression level in different cancer types based on GEPIA2 database. (b) Sema3D expression level in ccRCC tissues and normal tissues in the TCGA database and GTEx database. (c) Quantified Sema3D protein expression level by immunofluorescence staining in ccRCC tumor tissues and paired para-tumor tissues. (d) Immunofluorescence staining of Sema3D in ccRCC (400× magnification). Scale bars: 20 µm ( ^*∗*^*p* < 0.05,  ^*∗*^ ^*∗*^*p* < 0.01,  ^*∗*^ ^*∗*^ ^*∗*^*p* < 0.001, ns = not significant).

We investigated the level of Sema3D expression in pan-cancer and matching normal tissues in GEPIA2 database (Figure 2(a)). The Sema3D mRNA expression profile in ccRCC showed a lower expression level of Sema3D in tumor tissues compared with normal tissues (*p* < 0.001, Figure 2(b)), suggesting that low expression of Sema3D may be involved in the development of ccRCC. To further validate Sema3D expression in ccRCC, we performed immunofluorescence staining on the TMA of ccRCC specimens. As shown in Figure 2(d), Sema3D expression was mainly found in the extracellular matrix. Compared to 86 tumor specimens, we confirmed the Sema3D expression level was higher in paired para-tumor tissues (*p* < 0.001, Figure 2(c)).

### 3.2. The Relationship between Sema3D Expression and Clinical Data in ccRCC Patients

**Figure 3:**
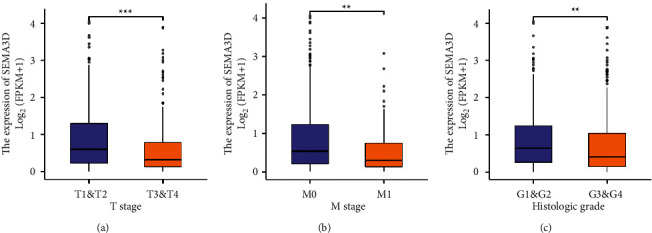
The association of Sema3D expression and clinical characteristics in clear cell renal cell carcinoma (ccRCC). (a) Tumor T stage. (b) Tumor M stage. (c) ISUP histologic grade ( ^*∗*^ ^*∗*^*p* < 0.01,  ^*∗*^ ^*∗*^ ^*∗*^*p* < 0.001).

The correlation between Sema3D expression and clinical data was then analyzed, and the grouping was in accordance with the 25% and 75% expression of Sema3D. The clinical characteristics of patients in TCGA database are presented in Supplementary Table S1. We found that patients with low Sema3D mRNA expression were significantly correlated with advanced *T* stage (*p* < 0.001), *M* stage (*p* < 0.004), and ISUP histologic grade (*p* < 0.002) (Figures 3(a)–3(c)).

### 3.3. Sema3D Is an Independent Biomarker of Prognosis in the Localized ccRCC Cohort

**Figure 4:**
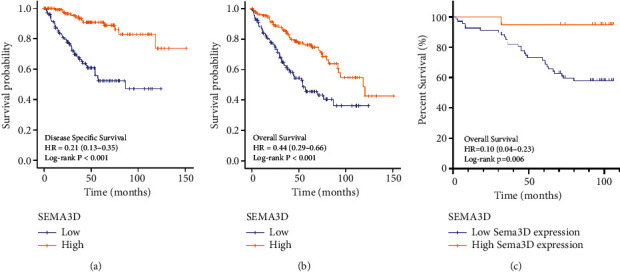
Kaplan–Meier survival analyses of clear cell renal cell carcinoma (ccRCC) patients stratified by the expression level of Sema3D. (a) Kaplan–Meier curves showing disease-specific survival (DSS) of patients in TCGA database. (b) Kaplan–Meier curves showing overall survival (OS) of patients in TCGA database. (c) Kaplan–Meier curves showing OS of patients in the local cohort.

Next, in the Kaplan–Meier survival curve and log-rank test, we examined the association between the Sema3D mRNA expression and survival in ccRCC patients. As shown in Figures 4(a)–4(b), the decreased Sema3D expression was significantly associated with poorer DSS (HR = 0.21, log − rank *p* < 0.001) and OS (HR = 0.44, log − rank *p* < 0.001). In the 86 patient cohorts of the TMA (Figure 4(c)), we validated the consistent survival risk that low expression of Sema3D was correlated with poor prognosis of OS (HR = 0.10, log − rank *p* < 0.006).

By Cox regression analysis in Table 2, we revealed the protective role of Sema3D expression in the survival outcomes of the ccRCC patient cohort. For all patients in the ccRCC cohort of TMA, the univariate Cox regression suggested that age at surgery (HR = 1.047, *p*=0.008), advanced tumor *T* stage (HR = 2.914, *p* < 0.001), advanced AJCC stage (HR = 7.128, *p* < 0.001), high Fuhrman grade (HR = 4.910, *p* < 0.001), large tumor maximal diameter (HR = 1.208, *p*=0.002), and low Sema3D expression (HR = 0.103, *p*=0.025) were associated with poor OS. Multivariate Cox regression included the factors that proved to be prognostic indicators in univariate analysis. Notably, Sema3D expression remained an independent prognostic factor of OS (HR = 0.126, *p*=0.044) in multivariate analysis, as well as age at surgery (HR = 1.069, *p*=0.001), AJCC stage (HR = 5.038, *p*=0.044), and Fuhrman grade (HR = 4.208, *p*=0.001). To describe the impact on the prognosis of Sema3D expression in localized ccRCC, we conducted a subgroup analysis by excluding the locally advanced and metastatic patients (T3 grade, lymph node metastasis, and distant metastasis). A total of 81 patients were enrolled in the subgroup analysis (Table 3). In the multivariate analysis, Sema3D expression level (HR = 0.125, *p*=0.043) and age at surgery (HR = 1.061, *p*=0.004) were significantly associated with OS in localized ccRCC, while the tumor *T* stage (HR = 1.786, *p*=0.333), Fuhrman grade (HR = 2.594, *p*=0.071), and tumor maximal diameter (HR = 1.079, *p*=0.323) were not assessed as prognostic indicators of OS. The reason of not evaluating *T* stage and tumor size as prognostic indicators was likely to be the correlation between Sema3D expression and *T* stage, which covered the intrinsic roles of *T* stage and tumor size in the survival outcomes.

Taken together, reduced Sema3D expression level was an independent predictor of poor prognosis for ccRCC patients, suggesting Sema3D in tumor tissue can be a potential prognostic biomarker for localized ccRCC patients.

### 3.4. Construction of PPI Network and Identifying Sema3D-Related Signaling Pathways

**Figure 5:**
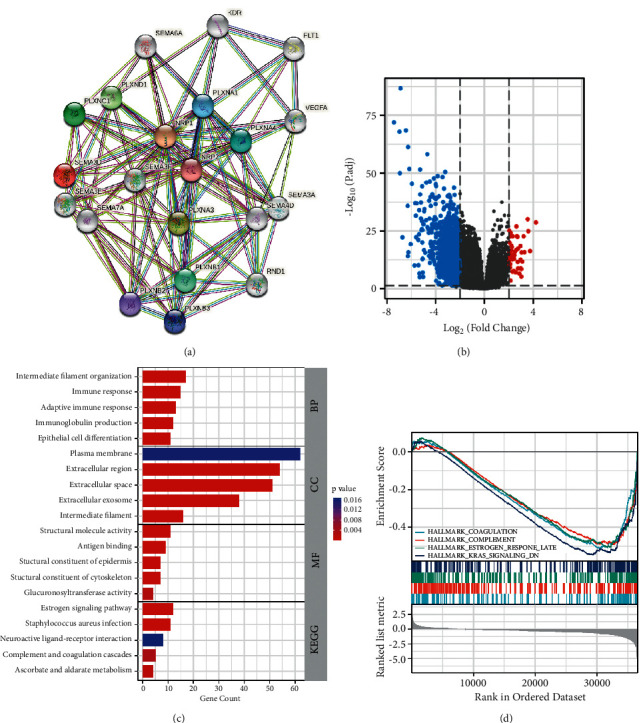
Differential expression and function enrichment analysis based on the RNAseq data of ccRCC from TCGA. (a) PPI network of proteins interacting with Sema3D. (b) Volcano plot showing differentially expressed genes (DEGs) based on Sema3D mRNA expression. The blue dots represent downregulated genes, and the red dots represent upregulated genes. (c) GO and KEGG function analysis of DEGs. The first group represents biological process (BP) group, the second group represents cellular component (CC) group, the third group represents molecular function (MF) group, and the last group represents KEGG group. (d) Pathway enrichment analysis of DEGs based on the hallmark gene sets.

The network plot in Figure 5(a) shows Sema3D and 20 encoding genes with interaction score >0.7. Apart from the major enrichment in semaphorin, neuropilin, and plexin family for neurogenesis, the interaction with kinase insert domain receptor (KDR), fms-related receptor tyrosine kinase 1 (FLT1), vascular endothelial growth factor A (VEGFA), and Rho family GTPase 1 (RND1) was notable as well. To further explore the potential mechanisms of Sema3D inhibiting tumor progression, based on the Sema3D expression level (<25% low group and >75% high group), we carried out a differential expression analysis of the RNAseq data in ccRCC from TCGA database. As can be seen from the volcano plot (Figure 5(b)), a total of 1,185 and 52 DEGs were found in the analysis. In the biological process (BP) section of GO analysis, DEGs were prominently enriched by intermediate filament organization, immune response, immunoglobulin production, and epithelial cell differentiation (Figure 5(c)). The function of plasma membrane, extracellular region, extracellular space, and extracellular exosome were described as the most enriched signaling pathways in the cellular component (CC) group. For the molecular function (MF) group, the structural molecule activity and antigen binding were highly enriched. In addition, the KEGG analysis indicated enriched neuroactive ligand-receptor interaction, *Staphylococcus aureus* infection, and complement and coagulation cascades. We also analyzed the DEGs in GSEA based on hallmark gene sets (Figure 5(d)). Four Sema3D-associated signaling pathways were revealed as follows: HALLMARK_COAGULATION, HALLMARK_COMPLEMENT, HALLMARK_ESTROGEN_RESPONSE_LATE, and HALLMARK_KRAS_SIGNALING_DN. The parameters for each GSEA are presented in Table 4.

### 3.5. The Landscape of Immune Infiltration in ccRCC and Sema3D Expression

**Figure 6:**
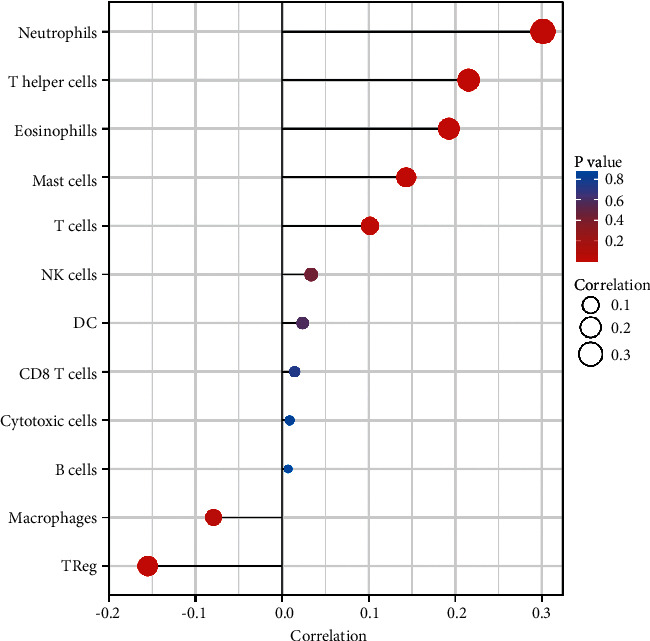
Correlation analysis of Sema3D expression with infiltrating immune cells calculated by Spearman correlation analysis.

To investigate the intrinsic role of Sema3D in the immune microenvironment in ccRCC, we then analyzed the correlation between Sema3D expression and tumor-infiltrating immune cells by identifying matching cell markers. Several subtypes of tumor-infiltrating immune cells were found correlated with the Sema3D expression level, including neutrophils (*r* = 0.301, *p* < 0.001), *T* helper cells (*r* = 0.215, *p* < 0.001), and eosinophils (*r* = 0.193, *p* < 0.001) (Figure 6).

## 4. Discussion

Sema3D gene, located on chromosome 7q21.11, generally induces the collapse and paralysis of neuronal growth cones, by secreting transmembrane protein, which could act as repulsive cues toward specific neuronal populations. Consistent with the Sema3D subcellular locations annotated from the Human Protein Atlas (HPA), by immunofluorescence staining, we detected secreted Sema3D in extracellular space and plasma membrane mostly. Unlike other semaphorins, family proteins exist primarily as membrane-bound forms, and class 3 semaphorins are secreted as soluble molecules [18]. Some research showed that Sema3D was correlated with familial Meniere disease, development of epicardium, and Hirschsprung disease [19–21]. Notably, previous studies also revealed involvement in carcinogenesis and potential mechanisms of Sema3D. Although one study indicated that samples of bladder cancer downregulated by Sema3D had a good overall survival prognosis [22], more studies tended toward the tumor suppressive role of Sema3D by experiment validation [23, 24]. However, the Sema3D protein expression and potential mechanisms have not been investigated in ccRCC and remained unclear.

In the present study, we included 86 ccRCC patients to investigate the protein expression of Sema3D in ccRCC. By analyzing the data from TCGA database and validating the results in the TMA samples, we found lower Sema3D expression in ccRCC tumor tissues than para-tumor tissues, and low Sema3D level was associated with poor prognosis in ccRCC patients. Bioinformatics analysis showed the correlation between low Sema3D level and advanced tumor stage. Our previous integrative genomic studies in ccRCC detected missense variants of Sema3D (amino acid change of D186 N) in both primary tumor and tumor thrombus from a patient with metastatic ccRCC. The shared mutation of Sema3D and annotation by PeCanPie suggested that Sema3D mutation might be an undiscovered driver mutation in ccRCC. We further analyzed the impact of Sema3D expression level on the development of ccRCC in this study. Overall, for patients with localized RCC, the ten-year probabilities of kidney cancer death, other cancer death, and noncancer death were 7%, 11%, and 22%, respectively [25]. Therefore, the exploration of prognostic biomarkers needs high confident long-term validation to initiate facilitating the translation of lab tests to the clinical setting. To date, molecular biomarkers for prognosis such as carbonic anhydrase IX (CaIX), vascular endothelial growth factor (VEGF), hypoxia-inducible factor (HIF), Ki-67, p53, p21 [26], PTEN, CXCR4 [27], and PD-L1 [28] have been investigated, but there has no recommended prognostic model of molecular biomarkers for localized ccRCC. In the subgroup analysis of the localized ccRCC patient cohort, we proposed Sema3D here as a novel prognostic biomarker. One would expect a feasible biomarker set with an acceptable predictive performance that can be detected by immunohistochemistry staining, based on which an advanced strategy of stratifying the risk in localized ccRCC may guide the follow-up and possible adjuvant therapy.

Hallmark gene sets are collections of refined gene sets derived from multiple MSigDB sets, describing a specific biological process and providing refined inputs for pathway enrichment analysis [29]. By identifying the DEGs of Sema3D, we demonstrated that these genes were primarily enriched in coagulation. GSEA also identified complement, estrogen response, and KRAS signaling as Sema3D-related signaling pathways. As the coagulation system is a vital component in the biology of neoplasms, the plasma level of D-dimer and fibrinogen reflecting the coagulation and fibrinolytic activities were likely to be correlated with high risks of tumor progression and metastasis in patients with RCC [30]. Moreover, one previous study in our medical center also proved that the extrinsic coagulation pathway participated in promoting the survival of circulating tumor cells (CTCs) in ccRCC [31]. However, the intrinsic role of Sema3D in coagulation still needs further validation. In terms of biological process in the GO consortium, Sema3D gene was involved in positive regulation of cell migration and cell differentiation [32]. After knocking down Sema3D mRNA expression with Sema3D-siRNA in colorectal cancer cells, Wang et al. found the capacity of cell migration was increased significantly [23], of which the underlying mechanism might be associated with the PI3K/Akt signaling pathway [33]. Li et al. included Sema3D in the immune-related hub genes, and Sema3D was identified as a protective factor of OS for patients with ccRCC [34]. To date, no research has revealed the link of immune cell infiltration and Sema3D expression in ccRCC. The immune infiltration correlation analysis in this study suggested several immune cells (neutrophils, eosinophils, and *T* helper cells) were highly associated with Sema3D in the tumor microenvironment. In our study, the function enrichment analysis also indicated immune response-related pathways played a role in ccRCC. However, the underlying mechanisms need further validation of experiment to provide stronger evidence.

## 5. Conclusions

In summary, our study identified the transmembrane transporter Sema3D as a tumor suppressor in ccRCC, and the Sema3D protein expression level was an efficient prognostic biomarker for localized ccRCC patients. We observed a significant correlation between Sema3D and coagulation, complement, estrogen response, and KRAS signaling. Higher expression of Sema3D was associated with better OS of ccRCC patients, suggesting that Sema3D can be a novel therapeutic target for ccRCC. Our study suggested that the interaction with coagulation system, KRAS signaling, and tumor neutrophil infiltration were the potential mechanisms of Sema3D serving as a tumor suppressor in ccRCC. However, the link of Sema3D and these mentioned pathways are still in need of further exploration.

## Figures and Tables

**Table 1:** Demographics and clinical characteristics of ccRCC patients according to the Sema3D expression.

Parameters	All patients (*n* = 86)	Low expression (*n* = 67)	Expression (*n* = 19)	*p* value
Age				
<60 years	44 (51.2)	35 (52.2)	9 (47.4)	0.842
≥60 years	42 (48.8)	32 (47.8)	10 (52.6)	
Gender				
Male	46 (53.5)	37 (55.2)	9 (47.4)	0.608
Female	40 (46.5)	30 (44.8)	10 (52.6)	
T stage				
T1	64 (74.4)	48 (71.6)	16 (84.2)	0.292
T2	18 (20.9)	15 (22.4)	3 (15.8)	
T3	4 (4.7)	4 (6.0)	0 (0)	
N stage				
N0	85 (98.8)	66 (98.5)	19 (100.0)	>0.99
N1	1 (1.2)	1 (1.5)	0 (0)	
M stage				
M0	84 (97.7)	65 (97.0)	19 (100.0)	>0.99
M1	2 (2.3)	2 (3.0)	0 (0)	
AJCC stage				
I/II	81 (94.2)	62 (92.5)	19 (100.0)	0.462
III/IV	5 (5.8)	5 (7.5)	0 (0)	
Fuhrman grade				
I/II	72 (83.7)	55 (82.1)	17 (89.5)	0.514
III/IV	14 (16.3)	12 (17.9)	2 (10.5)	
Median tumor maximal diameter (cm)	5.0	5.0	5.5	0.965

**Table 2:** Univariate and multivariate Cox regression analyses of OS in 86 enrolled ccRCC patients.

Covariates	Univariate analysis		Multivariate analysis	
	HR (95% CI)	*p* value	HR (95% CI)	*p* value
Age at surgery	1.047 (1.012 − 1.083)	0.008 ^*∗*^	1.069 (1.028 − 1.113)	0.001 ^*∗*^
Gender (male vs female)	0.871 (0.419 − 1.811)	0.711		
T stage (T1 vs T2 vs T3)	2.914 (1.707 − 4.974)	<0.001 ^*∗*^	1.213 (0.476 − 3.090)	0.686
AJCC stage (I/II vs III/IV)	7.128 (2.247 − 20.938)	<0.001 ^*∗*^	5.038 (1.043 − 24.344)	0.044 ^*∗*^
Fuhrman grade (1/2 vs 3/4)	4.910 (2.305 − 10.461)	<0.001 ^*∗*^	4.208 (1.814 − 9.764)	0.001 ^*∗*^
Tumor maximal diameter	1.208 (1.070 − 1.364)	0.002 ^*∗*^	1.083 (0.939 − 1.249)	0.271
Sema3D expression (low vs high)	0.103 (0.014 − 0.755)	0.025 ^*∗*^	0.126 (0.017 − 0.942)	0.044 ^*∗*^

**Table 3:** Univariate and multivariate Cox regression analyses of OS in 81 enrolled localized ccRCC patients.

Covariates	Univariate analysis		Multivariate analysis	
	HR (95% CI)	*p* value	HR (95% CI)	*p* value
Age at surgery	1.052 (1.013 − 1.091)	0.008 ^*∗*^	1.061 (1.020 − 1.105)	0.004 ^*∗*^
Gender (male vs female)	0.867 (0.394 − 1.910)	0.723		
T stage (T1 vs T2)	3.471 (1.567 − 7.686)	0.002 ^*∗*^	1.786 (0.552 − 5.781)	0.333
Fuhrman grade (1/2 vs 3/4)	3.943 (1.694 − 9.177)	0.001 ^*∗*^	2.594 (0.923 − 7.290)	0.071
Tumor maximal diameter	1.201 (1.050 − 1.373)	0.007 ^*∗*^	1.079 (0.928 − 1.254)	0.323
Sema3D expression (low vs high)	0.114 (0.015 − 0.845)	0.034 ^*∗*^	0.125 (0.017 − 0.934)	0.043 ^*∗*^

**Table 4:** Parameters in GSEA.

ID	Enrichment score	NES	*p* value	*p*.adj	*q* values
HALLMARK_COAGULATION	−0.513	−1.511	0.001	0.010^∗^	0.009
HALLMARK_COMPLEMENT	−0.492	−1.473	0.001	0.010^∗^	0.009
HALLMARK_ESTROGEN_RESPONSE_LATE	−0.507	−1.517	0.001	0.010^∗^	0.009
HALLMARK_KRAS_SIGNALING_DN	−0.547	−1.636	0.001	0.010^∗^	0.009

## Data Availability

The data used to support the findings of this study are included within the article.

## Abbreviations

RCC: Renal cell carcinoma
ccRCC: Clear cell renal cell carcinoma
WES: Whole-exome sequencing
Sema3D: Semaphorin 3D
TMA: Tissue microarray
TCGA: The Cancer Genome Atlas
GTEx: Genotype-Tissue Expression Project
DEGs: Differentially expressed genes
GO: Gene Ontology
KEGG: Kyoto encyclopedia of genes and genomes
DSS: Disease-specific survival
OS: Overall survival
HR: Hazard ratio
CIs: Confidence intervals
BP: Biological process
CC: Cellular component
MF: Molecular function
CTCs: Circulating tumor cells
HPA: Human Protein Atlas
CaIX: Carbonic anhydrase IX
HIF: Hypoxia-inducible factor
PD-L1: Programmed death ligand-1.

## Supplementary Materials

Supplementary Materials Supplementary Table S1: Patient characteristics in KIRC-TCGA database.
