# Supplementary material for: Transmembrane Transporter Sema3D Serves as a Tumor Suppressor in Localized Clear Cell Renal Cell Carcinoma
Source: J Oncol. 2022 Jun 30;2022:3204189. doi: 10.1155/2022/3204189 (PMC9262505; doi:10.1155/2022/3204189)
Supplement: Supplementary Materials — Supplementary Table S1: Patient characteristics in KIRC-TCGA database. [file 3204189.f1.docx]

**Supplementary Table S1.** Patient characteristics in KIRC-TCGA databse.

| Characteristic | levels | Overall |
| --- | --- | --- |
| n |  | 539 |
| T stage, n (%) | T1 | 278 (51.6%) |
|  | T2 | 71 (13.2%) |
|  | T3 | 179 (33.2%) |
|  | T4 | 11 (2%) |
| N stage, n (%) | N0 | 241 (93.8%) |
|  | N1 | 16 (6.2%) |
| M stage, n (%) | M0 | 428 (84.6%) |
|  | M1 | 78 (15.4%) |
| Pathologic stage, n (%) | Stage I | 272 (50.7%) |
|  | Stage II | 59 (11%) |
|  | Stage III | 123 (22.9%) |
|  | Stage IV | 82 (15.3%) |
| Gender, n (%) | Female | 186 (34.5%) |
|  | Male | 353 (65.5%) |
| Race, n (%) | Asian | 8 (1.5%) |
|  | Black or African American | 57 (10.7%) |
|  | White | 467 (87.8%) |
| Age, n (%) | <=60 | 269 (49.9%) |
|  | >60 | 270 (50.1%) |
| Histologic grade, n (%) | G1 | 14 (2.6%) |
|  | G2 | 235 (44.3%) |
|  | G3 | 207 (39%) |
|  | G4 | 75 (14.1%) |
| OS event, n (%) | Alive | 366 (67.9%) |
|  | Dead | 173 (32.1%) |
| DSS event, n (%) | Alive | 420 (79.5%) |
|  | Dead | 108 (20.5%) |
| Age, median (IQR) |  | 61 (52, 70) |
